# How are agency and autonomy understood in children’s palliative and end-of-life care?: A narrative review

**DOI:** 10.1177/26323524261460928

**Published:** 2026-07-16

**Authors:** Sydney Campbell, Lauren Delaney, Nika Rovensky, Ryan Kent, Amarens Matthiesen, Franco A. Carnevale, Mary Ellen Macdonald

**Affiliations:** 1 Division of Palliative Medicine, Department of Medicine, 3688 Dalhousie University, Halifax, Nova Scotia, Canada; 2 VOICE (Views on Interdisciplinary Childhood Ethics): The Childhood Ethics Project, 5620 McGill University, Montréal, Québec, Canada; 3 Faculty of Health Sciences, Queen’s University, Kingston, Ontario, Canada; 4 Ingram School of Nursing, 5620 McGill University, Montréal, Québec, Canada

**Keywords:** children’s palliative care, autonomy, agency, narrative review, childhood ethics

## Abstract

Public policy in Canada develops through deliberative democracy and, increasingly, public engagement. Children and youth (‘young people’) remain largely excluded from these processes however; this exclusion is especially evident in health arenas where two conceptual tools, autonomy and agency, are used to justify it. To date, there has not yet been an examination of how these concepts function in children’s palliative and end-of-life (P-EOL) care. This narrative review aimed to explore how autonomy and agency are constructed, conveyed, and employed in related literature. To situate and compare understanding of agency and autonomy, we used principles from Childhood Ethics. We scanned four databases using the search terms: young people; P-EOL care; agency; and autonomy. Studies were included if they were peer-reviewed, in English/French, and focused on young people’s agency and/or autonomy in P-EOL care. Exclusion criteria included an exclusive focus on neonates. Data were extracted from 52 sources focusing on study location, population, classification of P-EOL experiences, whether and how agency and autonomy were described, and barriers and facilitators to agency/autonomy expression. Analysis highlighted the prominence of autonomy in the literature in comparison to agency, and the conflation of these concepts. The mobilization of both concepts could bolster or thwart young people’s engagement in P-EOL care. Several modalities for expressing agency and autonomy were exemplified across the literature, including acute treatment decisions, research participation, decisions regarding sexuality and fertility, and prioritization of play. Overall, this study describes how interpretations of agency and autonomy can inform young people’s P-EOL care. Future research would benefit from prioritizing young people’s inclusion in P-EOL care research and generating communication-related training for clinicians.

## 1. Introduction

In Canada, a northern hemisphere high-income country with liberal traditions, deliberative democratic principles, laws, and regulations are central in the development of public policy, operationalized through a variety of tools, practices, and normative concepts (e.g., voting, public dialogue, consent, capacity).^1^ Though inclusion, equity, and justice are important values in and for policy development in Canada, several social groups remain excluded from contributing to policy debates and decisions that ultimately will impact their lives. For instance, children and youth (hereafter, young people) are not consistently involved in micro-, meso-, and macro-level policy development in many realms, even with regards to topics that directly concern them (e.g., bullying laws,^2^ education curriculum,^3^ city and transportation planning^4^).

One domain in which young people’s engagement is particularly lacking is palliative and end-of-life (P-EOL) care, including in contexts of policy and research.^5–7^ This lack of engagement is a moral infringement according to principles of justice and equity, and reinforces dominant discourses about childhood that overlook the capacities young people have to meaningfully contribute to concerns affecting them. Specifically regarding P-EOL care, this lack of engagement can result in services that are not tailored to young people’s needs and priorities.^7,8^ Young people have rights to receive care based on their best interests, as enshrined in the United Nations Convention on the Rights of the Child (UNCRC)^9^ and national rights-based frameworks (e.g., the Canadian Charter of Rights and Freedoms^10^). While we recognize the UNCRC’s pivotal role in promoting and enhancing necessary global policy dialogues around children’s rights, it endorses an understanding of childhood that is entrenched in Western, colonial representations of children as individuals in need of protection.^11^ Nevertheless, the dearth of opportunities for young people to participate in defining and operationalizing their best interests infringes on their rights.

Conceptual tools, such as social constructions of childhood^12^ and related ethical and legal concepts, can help surface whether and how young people are positioned as deserving of, and given access to, opportunities to be engaged.^13^ Agency and autonomy are commonly used to frame and justify young people’s inclusion – or lack thereof – in the context of healthcare.^14–16^ To date, no review has been conducted to make clear how these concepts are understood in P-EOL care, or how they can and do influence practice.^15^

We conducted a narrative review to better understand how agency and autonomy are constructed, conveyed, and employed in literature related to young people in P-EOL care. This review, conducted as part of a larger project on engaging youth in policymaking around P-EOL care, sought first to answer the question: How are the concepts of agency and autonomy being used in literature on young people’s end-of-life experiences? Following, and drawing on the Childhood Ethics framework, we sought to provide an analysis that answers: How should these concepts be used, moving forward?

## 2. Theoretical framework

We used the Childhood Ethics framework as the foundation for this project. This framework focuses on the moral dimensions of young people’s lives, seeing their participation in domains relevant to them as legitimate and valuable.^9,17^ Childhood Ethics is informed by a social constructivism lens, with the constructs of ‘childhood’ and ‘families’, alongside related concepts of ‘voice’, ‘engagement’, and ‘decision making’, understood as embedded in socio-political contexts.^17,18^ Central to Childhood Ethics is the concept of **agency**, defined as a young person’s capacity to act deliberately, speak for themselves, and actively reflect on their social worlds.^19^ Agency is positioned as an *inherent quality* of personhood that can be shaped, bolstered, or thwarted by social interactions across one’s lifespan, including at a person-to-person level and at a systems-level. Enabling a young person to realize/actualize their inherent agency requires an active investment in relationships to build trust, but it also requires authorities and ‘power brokers’ to critically assess systems and societal structures that work to inhibit young people’s agency realization or activation. Agency can manifest through three interrelated dimensions: aspirations, concerns, and capacities.^20^ *Aspirations* refer to the wishes young people have for themselves, for others, and for their environments/contexts. *Concerns* refer to areas of focus that a young person has due to experiencing or needing to worry about the potential for thwarted aspirations. *Capacities* refer to the diverse tools or abilities young people have to navigate their aspirations and concerns, and are positioned as expressive mechanisms (i.e., as opposed to a legalistic ‘capacity’ criterion associated with one’s ability to provide informed consent/refusal).

In refining agency, childhood scholars have critiqued dominant understandings of autonomy for impeding one’s ability to listen to and hear young people’s voices.^21^ **Autonomy** is conventionally understood as equated with one’s ability to ‘self-govern’. It is self-focused and self-directed, *acquired* throughout one’s development and *assigned* through formal recognition, for example through local legal standards. In contrast, Childhood Ethics scholars have proposed relational models of autonomy,^17,21^ derived from feminist ethics scholarship and attuned to the social embeddedness of persons that inform their identities.^15,22^ Seeing autonomy as relational allows for young people to be recognized and respected, even if not legally autonomous.^17^

Through using relational models of autonomy, clinicians and policymakers are directed to (re)define their legal and ethical responsibilities in patient (and public) interactions they undertake. Practically, this may involve reframing shared decision-making processes to be child-inclusive, developing new ways to think through ethical issues existing in the “messy grey zone”, and situating clinicians’ and policymakers’ own sense of autonomy in care and policy contexts.^15^ Understanding agency directs clinicians and policymakers to become more aware of how they conceptualize and position the ‘child’, who is owed these professional responsibilities and impacted by the legal and ethical ramifications. Combined, these concepts have practical implications for not only *what* clinicians and policymakers consider to be meaningful engagement processes of young people (as informed by relational autonomy), but also for *why* and *how* engagement is framed as important and necessary; that is, what rationales clinicians and policymakers can use in justifying child and youth inclusion in P-EOL care and policy. In this way, tapping into the agency of young people to highlight their unique and complex experiences and perspectives, combined with their shared humanity, can be a starting point.

## 3. Methodology and methods

### 3.1. Methodology

Given that agency and autonomy are multifaceted constructs shaped by ethical frameworks, clinical practices, and sociocultural assumptions, a narrative synthesis approach was chosen. Narrative synthesis enables integration across empirical, conceptual, and normative literatures, allowing for conceptual depth and reflexive critique, and providing insight into how knowledge evolves and might be advanced.^23,24^ A comprehensive search strategy was developed (see Appendix 1) with support from a university-based librarian to identify literature related to agency and autonomy in young people’s P-EOL care. Four electronic databases were searched: MEDLINE (OVID), PsycINFO (OVID), Sociological Abstracts, and CINAHL. Search terms focused on three key concepts: population (e.g., children, youth, young people), care context (e.g., end-of-life care, palliative care, assisted dying), and agency/autonomy (e.g., capacity, ability, autonomy, agency). Strategies were iteratively refined for sensitivity and comprehensiveness. The initial search yielded 8,806 records, which after de-duplication resulted in 7,706 unique articles screened for relevance.

### 3.2. Eligibility criteria

Eligibility criteria were determined a priori and refined through calibration exercises between the two reviewers (SC and LD). Empirical and conceptual sources were included if they were a) peer-reviewed manuscripts and academic texts (i.e. books, book chapters), b) in English or French, and focused on c) young people, defined broadly as children through young adulthood (approximately 3 years to 24 years of age), and d) concepts of agency or autonomy related to any aspect of EOL care, including palliative care, treatment withdrawal, management of life-limiting conditions, and assisted dying. Sources exclusively addressing newborns, infants, or toddlers were excluded, given the important differences that exist in thinking through agency and autonomy for these populations. We did not exclude sources based on any geographic criteria.

### 3.3. Screening process

**Figure 1.**
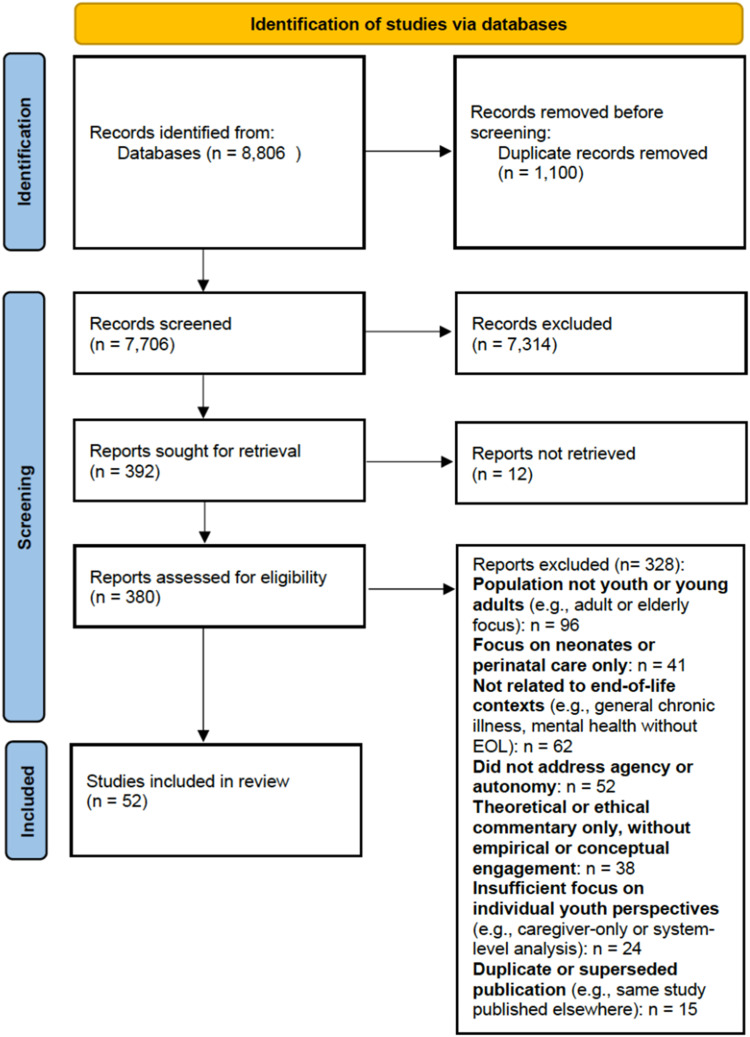
PRISMA^23^ flow chart for selection of literature.

Screening occurred in multiple stages to ensure consistency and reliability. An initial 10% subset of titles and abstracts was independently screened by both reviewers, with discrepancies resolved through discussion and regular calibration meetings. Once strong inter-reviewer agreement was established, the remaining records were divided for individual screening. Ultimately, 392 articles were retained for full-text review, with 380 successfully retrieved. Approximately 25% of full texts were double-reviewed to establish consistency. The final dataset included 52 articles (see Figure 1).^25^

### 3.4. Data extraction, quality assessment, and synthesis

Data extraction used Microsoft Excel with a structured form based on the study’s conceptual framework and objectives. Extracted data included study location, study population, classification of P-EOL experiences, descriptions of agency and autonomy, and barriers and facilitators to their expression and achievement. Methodological rigor, sample characteristics, conceptual clarity, and theoretical framing were critically assessed during extraction and synthesis, informing the weighting and contextualization of findings. Thematic analysis, guided by deductive categories from Childhood Ethics,^17^ identified patterns in how agency and autonomy were conceptualized, expressed, and constrained or facilitated across diverse P-EOL experiences.

## 4. Results

Details about source characteristics are outlined in Table 1.

**Table 1.** Characteristics of screened literature.

Source (N=52) Characteristic	Number	Details
**Type**		
Peer-reviewed articles	46	​
Book Chapters	5	
Books	1	
**Population Focus**		
Directly engaged young people	16	Literature explicitly involving young people, or drawing on their data
Indirect perspectives	36	Perspectives from parents/caregivers, healthcare providers, or conceptual/legal scholarship
**Design**		
Literature reviews	14	​
Qualitative studies	13	
Quantitative studies	2	
Mixed methods studies	2	
Case commentaries and normative analyses	14	
Legal analyses	3	
Conceptual analyses	3	
**Geographic Location**		
USA	16	​
UK	5	
The Netherlands and/or Belgium	4	
Switzerland	2	
Mexico	2	
Australia	2	
Italy	2	
Canada	1	
Jordan	1	
Sub-Saharan Africa (Kenya, Namibia, South Africa, Uganda)	1	
Norway	1	
Not specified or various	15	

In what follows, we begin by outlining how the concepts of agency and autonomy were conceptualized in the literature. Following, we examine how these concepts shaped young people’s P-EOL experiences, and how they could be recognized and manifested in practice.

### 4.1. Conceptualizations of agency and autonomy

#### 4.1.1. Defining autonomy

In the vast majority of the literature, references to autonomy were more prevalent than agency. Autonomy was primarily associated with competency, and cognitive or legal capacity. When a young person was deemed legally competent within a specific jurisdiction or clinical context, they were then seen to possess autonomy; in this sense, autonomy was presented as something that was acquired and bestowed. With the ‘possession’ of autonomy, young people were attributed with the ability to participate in decision making about their healthcare. Autonomy was connected with rights:^26–29^ either rights to privacy, freedom of choice, and freedom of action,^26–28,30^ or rights outlined in the UN Convention on the Rights of the Child.^27^ The potential of young people to have decisional autonomy was precipitated by their awareness of their best interests and ability to articulate and form preferences around those interests.^26,29–33^ Cultural and contextual considerations also informed how best interests were defined in a family unit, and the practical implications that followed, regarding elements of care like communication and decision making.^26,31,33–47^

##### 4.1.1.1. The acquisition of autonomy

Determining *whether* and *when* a young person ‘gained’ autonomy was a common inquiry. Many authors relied upon an age-based developmental interpretation of childhood in framing autonomy: whether a young person possessed autonomy and thus a capacity to consent, assent, or refuse treatment was seen to depend on their age and psychological development.^27,44,47–54^ Some authors outlined specific thresholds informed by neuroscience.^42,45,52^ These thresholds varied: some were set at age 14 years,^42^ 16 years,^45^ and mid-20s.^52^ Others framed age as a guiding factor.^43,55^

In contrast, a few articles were based upon an understanding of autonomy that was independent of age, placing greater emphasis on assessments of individual characteristics (e.g., conditioning, cultural influences, values, personal experiences) to determine who had sufficient capacity to make particular decisions.^50,53,56,57^ Some authors claimed models that de-centred age were particularly important for young people who had a life-limiting illness,^58,59^ especially in the context of health-related quality-of-life research, because their impending death “truncate [d] the burgeoning process of autonomy” and the age restriction became inappropriate.^59p. 3^

##### 4.1.1.2. Outlooks on autonomy

Several sources framed young people’s autonomy around the notion of self-governance and individualism, aligning with interpretations from adult contexts. Reasons for this included dominant discourses related to dying, which were focused on individual choice,^41^ and self-determination.^58^ In related arguments, legal capacity assessments sometimes acted as a rationale for evaluating young people’s autonomy. In turn, a young person had to show they had sufficient understanding of their illness and prognosis, along with “rationality” and an ability to communicate independent of others, in order to be deemed autonomous and capable of making decisions about their care.^28^ In some cases, this narrow definition led to the conclusion that children were incapable and thus non-autonomous because of their dependence on others, developing capacities, and lack of independent rationality.^60^

In contrast, many sources framed young people’s autonomy in a relational manner.^60^ Shared decision making approaches took priority in these sources as it allowed for the development of “a sense of mutual trust and respect between the child, caregiver and health professional”^36p. 531^ and for young people’s role in their own care to be included.^31,46^ Accordingly, the care circles of young people were seen to play a vital part in making decisions.^42,50,55^ Parents were particularly vital because of their familiarity with and connection to their child,^61–64^ including awareness of their best interests.^26,34,35,65^ Parents were especially important when a child’s health condition resulted in a loss of capacity to communicate.^54,66^ In a survey of young people with advanced cancer, results indicated that while young people did engage in collaborative decision making, they tended to be satisfied with shifting responsibility to parents and healthcare providers.^54^

###  4.1.2. Defining agency

Agency was explicitly referred to in eight of the 52 sources, and primarily in articles published over the last decade.^38,51,57,59,60,66–68^ The only source published earlier was Walter and Ross^60^ who used the term ‘agent’ to refer to a person to whom autonomy is assigned.^60^ A summary indicating how “agency” was used and/or defined in all eight articles is available in Table 2.

**Table 2.** Exploring the definitions and uses of agency in screened literature.

Author	Year	Definition or Use of Agency
Anath et al.^62^	2021	For adolescents and young adults, **agency** entailed being directly involved in conversations with their healthcare teams and being kept “in the loop” (pg. 494).
Badarau et al.^46^	2019	Young people have been seen as “moral **agents**” who are “capable of making decisions regarding their own health” in ways that align with their capacities (pg. 63).
Bluebond-Langner et al.^61^	2021	Young people are “active **agents**” with views that develop socially, through interpreting the behaviour of others and responding to those interpretations. They interact with the world and make judgements from their own observations “inside and outside of themselves” (pg. 1).
Bristowe et al.^63^	2024	Initially directing conversation and questions towards a young person, and encouraging their own response, is how a clinician can protect young people from a loss of **agency** (pg. 385). This involves using forms of communication that align with a young person’s understanding.
Ekberg et al.^60^	2022	In this article, **agency** is understood to be co-produced and co-constructed through social interactions that young people have with others. For a child to be considered a “social **agent**”, it requires recognizing their capacities and competency (pg. 568). In healthcare spaces in particular, sequences of talk (in a conversation) inform how this co-construction unfolds.
Fay et al.^35^	2021	**Agency**, according to the conceptualization advanced in this article by Gramsci (2005), is defined as “the ability to act both individually and collectively in the societal transformation brought about by the individual, and to how the individual is transformed by social and political structure” (pg. 2). Pediatric patients exercise **agency** relationally, with their family and healthcare providers. In pediatric contexts, the **agency **of the patient, parents (themselves and as proxies), and providers are “in play”, along with a collective **agency **existing at the plexus of these intersecting agencies (pg. 9).
Rahimzadeh et al.^48^	2015	Respecting young people’s **agency** can be achieved by “lending primacy to [their] voices” (pg. 3) and “rendering their continued absence in [pediatric palliative care] an antiquated protectionist argument of the past” (pg. 5).
Walter & Ross^50^	2013	**Agent** is a person who possesses autonomy.

In the more recent articles, there was an explicit recognition of children’s social position in expressions of what agency meant, and the implications of this term for young people’s role in their care.^38,51,59,66^ In addition to these articles, other articles implicitly touched on components of agency as articulated in our theoretical framework, specifically outlining how young people’s *concerns*, *aspirations*, and *capacities* were understood or expressed.

#### 4.1.2.1. Concerns

Concerns ranged from young people being worried about being left out of conversations and lacking information (including about medical decisions), which could be experienced as disrespectful,^57,69^ to concerns about being sedated throughout the end of their life, thereby rendering the young person unable to express their wishes and infringing on their “dignity”.^57^ Young people with life-limiting illnesses highlighted changes to their social engagements (including school attendance) as a pronounced loss in their lives.^48,68,69^ As they neared EOL, they could grieve losses, including loss of independence,^54,68,69^ and changing needs.^48,68,69^ Many authors framed the potential for physical, psychosocial, and existential suffering as a source of worry.^31,69,70^ For instance, in a survey of young people with HIV and AIDS, 71% indicated that they feared dying painfully.^70^ Further, young people’s position as inherently social was reflected in the concerns they had, wherein they could grieve for the suffering and impending loss that their families or care circles faced as a result of their health experiences.^32,48,69^

#### 4.1.2.2. Aspirations

Several authors referred to young people’s aspirations with regards to their involvement in their care, using descriptions of their “wishes”, “needs”, “wants”, “hopes”, and “preferences”.^32,34,35,57,63,65^ One of the key aspirations associated with life-limiting illnesses was being able to participate and express agency by being actively engaged and informed.^32,52^ For some, transparent communication with their team allowed them to have a sense of control in discussions about their care,^34,70^ death, and legacy.^53,57,70^

Some young people wanted their parents to be involved,^33,63^ reflecting their desire to shape their care. Authors associated parental involvement with a more accurate interpretation of the “voice of the child,”^61p. 450^ and their best interests and aspirations.^35^

Another key aspiration for young people was normalcy. Young people wanted to remain connected to the social worlds around them by establishing meaningful relationships,^46,71^ being physically active,^69^ having opportunities to play,^72,73^ or by being relieved of pain, symptoms, and hospitalizations.^34^ They also wanted to develop their own goals,^46^ and live “in the moment”.^69p. 326^

#### 4.1.2.3. Capacities

Finally, young people’s capacities were a core focus in many of the sources.^26,38,53,65,67^ Some authors spoke of young people’s ‘capacity’ as extending beyond an ability to make decisions to, instead, include their capacities to confront challenging moral issues. For instance, children displayed agency by “interpreting the behaviour of others and acting on the basis of those interpretations”^38p. 1^ responding to what was the ‘right’ thing to do. An example is situations in which a young person had gained awareness that they were dying despite being given partial information, and then acting to maintain hope for their family by ensuring their own awareness remained unspoken.^34^

### 4.2. How agency and autonomy shaped palliative and end-of-life experiences

Notwithstanding how agency and autonomy are unique concepts, both informed whether and how young people were engaged in their P-EOL care. The mobilization of these concepts could bolster or thwart their engagement in their care as outlined below.

#### 4.2.1. Communication by clinicians and parents

In contexts in which agency and/or autonomy were recognized as applicable to a young person, the practice of direct and honest communication by both clinicians and parents was a means to bolster young people’s engagement. In many articles, young people consistently emphasized the importance of being fully informed about their condition, prognosis, and treatment options so as to meaningfully participate in their care.^34,39,63,67^ Receiving clear and comprehensive information was a means to achieve and recognize their capacity for engagement.^34,39,63,67^ Researchers recommended that clinicians affirm their understanding of youth-specific needs and tailor communication based on an individualized assessment of each young person’s competency and capacities.^43^ Sensitivity, compassion, and respect for young people’s dignity and self-determination were identified as critical provider attitudes that enabled meaningful engagement and recognition of agency.^43^ Likewise, when parents engaged in open, honest dialogue with their children, delivered in a sensitive and attentive manner that promoted recognition of a young person’s agency, young people were better able to express their preferences and participate in decision making.^37,63,64^ Parents who respected their child’s right to know, even amidst their own fears and hopes, created spaces for autonomy and agency to flourish.

Conversely, withholding critical health information from young people (e.g., because of beliefs about their lack of autonomy and/or agency) thwarted potential for meaningful engagement. Across the sources it was evident that when parents or healthcare teams did not fully disclose prognostic information, young people were deprived of opportunities to reflect, adjust, and engage in their care decisions.^26,44,54,63,64^ The act of withholding information limited young patients’ ability to plan for the future, form personal reflections, and engage authentically in decision-making processes.^26,63,64^

The implications of (non)disclosure could be significant. Young people kept unaware of their prognosis were more likely to adopt passive roles in their care.^40^ In contrast, youth who were engaged in open communication were able to establish trust and exercise their agency,^64,67^ and enhance their emotional and social adjustment.^26^ Feeling understood, including recognition of personal beliefs and treatment preferences, was key to feeling respected in shared decision making.^67^ Notably for young people who chose to defer decisions to parents, respecting non-participation can also honor agency and autonomy.^63,67^

As a response to communication challenges, several authors advocated for specialized training for healthcare providers working in children’s P-EOL care settings.^27,32,33,43,53,55,74^ Literature indicated that without specific, continued preparation, even seasoned providers could struggle with effective communication, assessment of capacity, and facilitation of young people’s involvement.^32,33,43,55,74^ To meaningfully engage young people, authors highlighted that clinicians must have strong knowledge of their individual needs and interests, including an understanding of how best to involve them.^27,43,53^ Often, this involved challenging assumptions about young people’s agency and autonomy, particularly for young people with disabilities where biases could prevent consideration of their potential for meaningful participation.^27^ It could also involve having clinicians build their technical communication skills and promote their self-awareness, compassion, and ethical reflection, essential qualities for supporting autonomy and emotional wellbeing in youth.^43^

#### 4.2.2. Navigating hope

The role of hope emerged as a distinct and powerful factor informing whether young people’s agency and autonomy were bolstered or thwarted. Across multiple sources, maintaining hope (especially curative hope) was seen as essential to protecting their spiritual and emotional well-being.^33,37,40,48,63,74^ Whereas death in adult context is often framed as an event requiring planning, autonomy, and choice,^41^ parents of young people with life-limiting illnesses saw planning for death as incompatible with their child’s inherent drive to live. Instead, parents emphasized the importance of sustaining a “combative spirit,” choosing to focus on living in the present and avoiding discussions of prognosis or death.^33,40,41,48^ This emphasis on hope was informed by healthcare providers’ attitudes and clinical approaches too.^41,75^ Many providers saw themselves as guardians of hope, expressing fears that fully disclosing the extent of a child’s deterioration would extinguish the family’s morale and erode their coping.^26,41,43,44^ As such, prioritizing hope could result in avoidance in engaging young people in important EOL conversations and care-related decision.^35,44,75^

Alternatively, articles that emphasized the importance of advance-care planning (ACP) with young people saw this tool as a way to reframe hope. For these authors, ACP allowed simultaneous conservation of hope, protection of agency and autonomy, and an opportunity for engagement. ACP provided a structured process through which young people could express their values, articulate their care preferences, and exert meaningful influence over their EOL experiences^30,32,35,36,43,44,46,55,75^ and interpretations of living and dying well.^40,46,48,57,75^ When ACP was initiated early in the illness trajectory when young people were still capable of reflective participation, it provided space for ongoing dialogue to honor shared visions and values.^32,33^ Doing so facilitated better understanding of young people’s wishes for their care^32,33,36,39,44,46–48,53,54,57,63,70,71,74^ that could be family-inclusive.^70,71^ Rather than undermining hope, well-conducted ACP was seen to foster “hope-filled” communication, balancing realistic information about prognosis with encouragement toward meaningful, achievable goals.^44^ This reframing was seen to allow young people to maintain emotional resilience and foster a sense of agency, even as they faced EOL realities.

#### 4.2.3. Legal positions vis-à-vis consent and capacity

Across some sources, legal debates and regulations around consent and decision-making capacity were used to interpret young people’s agency and autonomy. Some positioned autonomy as a *positive right*, applicable to every individual regardless of age.^26^ Likewise, participation rights, especially those outlined in the UNCRC, were seen to be an important indication of a state’s obligations to ensure young people are able to participate in and express their views about areas that impact their lives, including healthcare decisions.^27^ In contrast, others positioned autonomy as a *negative right* connected to privacy, wherein states were seen to be guarantors of personal privacy and could only override autonomy when there was a compelling state interest.^28^

In some contexts, laws imposed rigid age thresholds that restricted young people’s participation in critical decisions.^53,62^ For instance, in many jurisdictions, minors (under the age of 18) are presumed not to have full legal competence to make independent medical decisions.^52,58^ Legal frameworks, such as the U.S. age of majority laws and the Family Law Reform Act in England and Wales, delineate a sharp divide (age of 18 years); such delineations fail to account for the potential of functional capacity demonstrated by some young people.^27^ Thus, even when young people demonstrate capacity, healthcare providers often defer to parents, requiring parental authorization before engaging young people directly in conversations about prognosis, treatment options, or research participation.^40,49^ This deference reflects both medico-legal caution and entrenched cultural norms about parental responsibility and protection, thereby limiting the realization of youth agency.

### 4.3. Recognition of agency and autonomy in practice

Several modalities were described in the literature that acknowledged and enabled young people’s agency and/or autonomy. Below we share a few examples.

#### 4.3.1. Treatment and care decisions

Vince and Petros^56^ reported a case of a 14-year-old boy who was admitted to the PICU with acute chronic respiratory failure. The boy’s respiratory function deteriorated despite extended mechanical ventilation. A decision was made jointly by the care team and parents that aggressive intensive therapy was futile and that withdrawal of therapy was the best option. A discussion ensued about whether the boy should be woken up to inform him of his terminal condition, express his choices around his death, and say goodbye to his family. While ultimately the decision to forego awakening the child was made, the discussion of the clinical team around whether to awaken the boy indicated the complexity of interpreting how best to respect the autonomy and agency of a young person at the end of their life.^56^

Additionally, three articles^50,57,76^ discussed the permissibility of engaging young people in decisions about medically-assisted dying. Bollen and colleagues,^76^ in particular, focused on the permissibility of organ donation decisions for young people who chose to die by an assisted death in the Netherlands and Belgium—two countries where assisted dying is legal for those under 18 years of age, though restricted by adult involvement to varying degrees in both countries and by complex legal processes. Interpretations of autonomy, in these contexts, already permit eligible young people to consent both to an assisted death and to being an organ donor, so researchers argue that having these decisions made in tandem would be odd to overlook or deny.^76^

#### 4.3.2. Research participation and involvement

Young people’s involvement in research, particularly as participants, was described in the literature as a modality through which young people could express their aspirations, concerns, and capacities.^49,59,75^ For instance, in a study examining pediatric patients’ perspectives on EOL care preferences, findings revealed that patients considered participating in a phase 1 study because “it could benefit another, although it might harm self”;^75, p. 9153 ^in other words, participating is what engaged young people feel is the ‘right’ thing to do.^75^ Yet, a systematic review of qualitative research conducted ‘with’ (instead of ‘on’) young people found there to be a paucity of relevant literature, and positioned it as an “emerging and promising field” important for the future of children’s palliative care.^77^

#### 4.3.3. Sexuality and fertility

A modality to support young people’s agency related to sexuality, sexual identity, and fertility was highlighted in two papers. Young people – regardless of, or because of, their disabilities and/or terminal diagnosis, may have concerns about their sexuality, achieving sexual milestones, and how their illness could affect their fertility.^48,52^ Opportunities to discuss sexual and reproductive health concerns and aspirations could be important for supporting their agency.^48,52^

#### 4.3.4. Play

Finally, play is an important facet of how young people navigate the world, express themselves, and is a right outlined in the UNCRC. Young people with life-limiting illnesses may seek opportunities to engage in play and may use play to express concerns, aspirations, and capacities.^66,72,73^

## 5. Discussion

This review is the first to examine how the concepts of autonomy and agency have been understood and applied in P-EOL care to justify and shape young people’s engagement (or lack thereof) in related decisions and discussions. In what follows, we situate our findings in existing literature and draw out important reflections related to: the relationship between autonomy and agency; the role of hope as related to these concepts; and limitations of our study along with future directions.

### 5.1. Examining the relationship between autonomy and agency

Through our review, the complex relationship between autonomy and agency became evident. We found that both the terms are used to describe where a young person ‘fits’ into decisions about their care (including in P-EOL contexts) and, as a result, may be conflated or used interchangeably. When agency was mentioned briefly or without analytic depth,^51,60^ this overlap was more challenging to detangle. Where possible, we attempted to distinguish these concepts by returning to our definitions that recognize agency as innate and autonomy as acquired. Overall, we found that autonomy continues to be a more prominent concept than agency to frame, direct, and critique young people’s engagement in P-EOL care. Yet, results also indicate that agency is emerging in recent years, perhaps cross-pollinated by childhood studies where the topic has been burgeoning over the last two decades.^78^

In healthcare and health policy settings, respecting patient autonomy has historically been considered central for medical ethics, provider training, and clinical practice^79,80^ including in pediatric care.^81^ Yet the concept of autonomy was derived from adult healthcare spaces, and there are several concerns that arise when borrowing or transferring concepts from adult discussions into the pediatric context.^82^ Most clearly, young people often navigate the world - and especially healthcare spaces - relying heavily upon their care circles and the sociopolitical, legal, and cultural systems they exist within; in other words, young people are inherently relational.^17^ And indeed, “dependency is an inherent feature of being human”.^81p. 5, 83^ Therefore, placing the goal posts for young people’s engagement (as with any person’s engagement) on the expectation that they must be ‘independent’ or ‘autonomous’ is unrealistic^81,83^ and unfair, setting them up to never being meaningfully engaged. One thing that is agreed upon by most scholars: relational, inter-generational models of agency and/or autonomy are required to move the needle forward in discussions about including young people in spaces that impact their lives,^15,78,84–87^ and to ensure their best interests are recognized and acknowledged in child-inclusive ways.^17^ These relational models acknowledge the complex nature of children’s healthcare considering the embodied aspects of shared decision making.^15^ This is especially salient in children’s palliative and EOL research, policymaking, and practice, where reliance on parents, family, healthcare providers, and community is perceived as central to high quality care.^81^

### 5.2. The role of hope in P-EOL care

Hope emerged in this review as a complex, dynamic force within P-EOL contexts. Hope is a central theme in how young people, parents, and clinicians navigate dying, with implications for agency, autonomy, and participation in care decisions. The literature conveys that hope is not static but undergoes transformation throughout the illness trajectory, shifting from a hope for cure to hopes for comfort, connection, and legacy.^44,46,48^ Moreover, families and clinicians play a key role in maintaining and preserving hope for the purposes of emotional resilience. Often this is associated with a focus on protection, which can then override opportunities for engaging young people and can lead to ethical tensions regarding medical decision making between patients, families, and their healthcare teams.^26,34,35,40,41,63,75^

Importantly, the literature opens the possibility for honest communication to bolster hope. Sources describe how hope can evolve when prognostic information and care plans are communicated with sensitivity.^88,89^ Both young people and their parents may shift from hoping for a cure to hoping for a good death, for comfort, or for meaningful final experiences.^90–93^ Tools like *Voicing My Choices* have been shown to support this transformation, helping youth articulate their emotional, spiritual, and existential goals.^33,44^ In these contexts, hope becomes future-oriented, not necessarily tied to survival, but to values, memory-making, and dignity. These are important characteristics when thinking about how best to support young people’s pursuit of respect for their agency and autonomy when they are facing a life-limiting illness.

### 5.3. Limitations and gaps

#### 5.3.1. Limitations

A limitation of our study is that our language and database limitations may have caused the exclusion of relevant literature (e.g., in other languages and grey literature). Further, our search strategy that focused on agency and autonomy may have missed differently framed but related concepts (e.g., resilience, dignity, coping). While our work and defined concepts may help researchers to consolidate terminology when writing about these topics, future integrative reviews could expand the conceptual landscape of youth participation at end of life to include additional concepts. In addition, future work could move beyond a Western orientation to think about young people’s agency, autonomy, and engagement in other sociocultural and geographic contexts.

#### 5.3.2. Future research

Of the 52 sources we reviewed, the majority reflected the views of parents, clinicians, or scholars. Only 16 explicitly engaged children and youth or drew from studies that included their perspectives; this underrepresentation of young people raises concerns about adult-centered knowledge production and the frequent reliance on proxy interpretations of youth preferences.^38,61^ This limitation results in an incomplete picture of how young people experience and express agency at the end of life^15^ and indicates a crucial area for continued research.

Further, this critique also applies to the conceptualization of constructs examined in this study. Agency and autonomy are largely adult-conceived and would benefit from being explicitly child- and youth-informed. Furture research would also require a nuanced lens for examining how agency and autonomy are understood by, and positioned in literature pertaining to, newborns, infants, and toddlers, along with those who communicate differently (e.g., young people who are non-verbal). Currently, priority is often granted to young people who have acquired linguistic comprehension, with a tendency to map cognition to the development of language expression. In these cases, the ‘preverbal’ and ‘non-verbal’ young person can be deemed uninterpretable by adults, and so their views (and by extension their agency and autonomy) are deprioritized, which aligns with how many treat and position other life forms without human-identifiable verbal communication capacities.^94^

To authentically capture how young people experience and express agency in P-EOL care, research should make use of participatory, child-centered methodologies that use visual, narrative, play-based, or assistive methods to support diverse expressions of agency.^66,72,73^ These approaches are especially important for young people whose agency and autonomy are often underestimated or overlooked. Such research should also examine how structural factors such as disability, race, Indigeneity, gender identity, and socioeconomic status shape EOL experiences and opportunities for participation, areas that remain underexplored in the current literature. This could, for instance, involve applying ‘intersectionality’ as an additional lens through which experiences in P-EOL care contexts are understood and related narratives about agency are further developed in socioculturally attuned ways. Practically, these studies should be shaped, informed, and led by these communities to align with their aspirations, goals, and preferences.

An additional area for future investigation is the training of healthcare providers in youth-centered P-EOL care. Many clinicians report lacking the skills or support needed to facilitate ethically grounded communication that effectively and respectfully invites young people into discussions.^32,43^ Best practices emphasize developmentally-informed communication training, legal and ethical education, and trauma-informed approaches.^27,33^ Interventions such as role-play, guided reflection, and structured tools have shown promise in enhancing clinician capacity to support youth engagement.^33,53^ However, few programs have undergone rigorous evaluation, and their long-term impact remains unclear.

## 6. Conclusion

This review is the first conceptual interrogation of how the concepts of autonomy and agency have been defined and mobilized in literature about young people’s P-EOL care, revealing important policy and practice gaps and implications. This work highlights the importance of recognizing the relational understandings of childhood in research about the lives of young people, while simultaneously acknowledging the knowledge gaps that exist as a result of limited work that directly engages young people as participants and as partners. Moving forward, scholars have a responsibility to ensure conceptual clarity for the terms used (whether autonomy or agency) and make space for the ethical and meaningful inclusion of young people in practice, policy, and research, including at the end of their lives where this aligns with their interests and aspirations.

## Appendix 1

### Search terms

#### PUBLISHED/PEER-REVIEWED literature

All* searches ran and imported into Covidence on Oct 2, 2024 (*except CINAHL, which was Oct 3, 2024)

**Table A1.** MEDLINE at OVID.

Line	Search	Results
1	(child or children or youth or “young people” or “mature minor” or “mature minors” or minor or minors or “young adult” or “young adults” or pediatric or paediatric or adolescent or adolescents).mp. [mp=title, book title, abstract, original title, name of substance word, subject heading word, floating sub-heading word, keyword heading word, organism supplementary concept word, protocol supplementary concept word, rare disease supplementary concept word, unique identifier, synonyms, population supplementary concept word, anatomy supplementary concept word]	4834143
2	Young Adulthood/or Adolescent/or Child/or Young Adult/	3730849
3	1 or 2	4834143
4	(“end of life” or “medical assistance in dying” or “medical aid in dying” or (medical adj3 dying) or “palliative care” or “withdrawing treatment” or “treatment withdrawal” or “withdraw treatment” or “treatment cessation” or euthanasia).mp. [mp=title, book title, abstract, original title, name of substance word, subject heading word, floating sub-heading word, keyword heading word, organism supplementary concept word, protocol supplementary concept word, rare disease supplementary concept word, unique identifier, synonyms, population supplementary concept word, anatomy supplementary concept word]	133753
5	Palliative Care/or “End of Life Care”/or Withholding Treatment/or Euthanasia, Active, Voluntary/or Euthanasia, Passive/or terminal care/or right to die/or terminally ill/or hospice care/	112429
6	4 or 5	156676
7	(capacity or capacities or ability or abilities or autonomy or autonomous or agency or agent or agential or engage*).mp. [mp=title, book title, abstract, original title, name of substance word, subject heading word, floating sub-heading word, keyword heading word, organism supplementary concept word, protocol supplementary concept word, rare disease supplementary concept word, unique identifier, synonyms, population supplementary concept word, anatomy supplementary concept word]	2823542
8	Personal Autonomy/or Agency/or decision making/or patient participation/or choice behavior/	175991
9	7 or 8	2957511
10	3 and 6 and 9	3199

**Table A2.** APA PsycInfo at OVID.

Number	Search	Results
1	(child or children or youth or “young people” or “mature minor” or “mature minors” or minor or minors or “young adult” or “young adults” or pediatric or paediatric or adolescent or adolescents).mp. [mp=title, abstract, heading word, table of contents, key concepts, original title, tests & measures, mesh word]	1322893
2	Young Adulthood/or Adolescent/or Child/or Young Adult/	9783
3	1 or 2	1325059
4	(“end of life” or “medical assistance in dying” or “medical aid in dying” or (medical adj3 dying) or “palliative care” or “withdrawing treatment” or “treatment withdrawal” or “withdraw treatment” or “treatment cessation” or euthanasia).mp. [mp=title, abstract, heading word, table of contents, key concepts, original title, tests & measures, mesh word]	26731
5	Palliative Care/or “End of Life Care”/or Withholding Treatment/or Euthanasia, Active Voluntary/or Euthanasia, Passive/or Terminal Care/or Right to Die/or Terminally Ill/or Hospice Care/	19488
6	4 or 5	29026
7	(capacity or capacities or ability or abilities or autonomy or autonomous or agency or agent or agential or engage*).mp. [mp=title, abstract, heading word, table of contents, key concepts, original title, tests & measures, mesh word]	867688
8	“Independence (Personality)”/or Autonomy/or Agency/	131097
9	7 or 8	957745
10	3 and 6 and 9	860

### Sociological Abstracts

(child or children or youth or “young people” or “mature minor” or “mature minors” or minor or minors or “young adult” or “young adults” or pediatric or paediatric or adolescent or adolescents).

#### AND

(“end of life” or “medical assistance in dying” or “medical aid in dying” or (medical adj3 dying) or “palliative care” or “withdrawing treatment” or “treatment withdrawal” or “withdraw treatment” or “treatment cessation” or euthanasia)

#### AND

(capacity or capacities or ability or abilities or autonomy or autonomous or agency or agent or agential or engage*) Results: 3251.

### CINAHL

(child or children or youth or “young people” or “mature minor” or “mature minors” or minor or minors or “young adult” or “young adults” or pediatric or paediatric or adolescent or adolescents)

#### AND

(“end of life” or “medical assistance in dying” or “medical aid in dying” or (medical adj3 dying) or “palliative care” or “withdrawing treatment” or “treatment withdrawal” or “withdraw treatment” or “treatment cessation” or euthanasia)

#### AND

(capacity or capacities or ability or abilities or autonomy or autonomous or agency or agent or agential or engage*) Results: 1496.
